# Aspirin has little additional anti-platelet effect in healthy volunteers receiving prasugrel

**DOI:** 10.1111/j.1538-7836.2011.04450.x

**Published:** 2011-10

**Authors:** P D M LEADBEATER, N S KIRKBY, S THOMAS, A-R DHANJI, A T TUCKER, G L MILNE, J A MITCHELL, T D WARNER

**Affiliations:** * Department of Cardiothoracic Pharmacology, Imperial College London, National Heart and Lung Institute; † The William Harvey Research Institute, Barts and The London School of Medicine & Dentistry, Queen Mary University of LondonLondon, UK; ‡ Departments of Pharmacology and Medicine, Vanderbilt University, NashvilleTN, USA; § The Ernest D Cooke Clinical Microvascular Unit, St Bartholomew’s HospitalLondon, UK

**Keywords:** aspirin, PGI-M, platelets, prasugrel, TX-M

## Abstract

**Summary:**

*Background:* Strong P2Y_12_ blockade, as can be achieved with novel anti-platelet agents such as prasugrel, has been shown *in vitro* to inhibit both ADP and thromboxane A_2_-mediated pathways of platelet aggregation, calling into question the need for the concomitant use of aspirin. *Objective:* The present study investigated the hypothesis that aspirin provides little additional anti-aggregatory effect in a group of healthy volunteers taking prasugrel. *Study participants/methods:* In all, 9 males, aged 18 to 40 years, enrolled into the 21-day study. Prasugrel was loaded at 60 mg on day 1 and maintained at 10 mg until day 21. At day 8, aspirin 75 mg was introduced and the dose increased to 300 mg on day 15. On days 0, 7, 14 and 21, platelet function was assessed by aggregometry, response to treatments was determined by VerifyNow™ and urine samples were collected for quantification of prostanoid metabolites. *Results:* At day 7, aggregation responses to a range of platelet agonists were reduced and there was only a small further inhibition of aggregation to TRAP-6, collagen and epinephrine at days 14 and 21, when aspirin was included with prasugrel. Urinary prostanoid metabolites were unaffected by prasugrel, and were reduced by the addition of aspirin, independent of dose. *Conclusions:* In healthy volunteers, prasugrel produces a strong anti-aggregatory effect, which is little enhanced by the addition of aspirin. The addition of aspirin as a dual-therapy with potent P2Y_12_ receptor inhibitors warrants further investigation.

## Introduction

The use of dual anti-platelet therapy comprising aspirin and a P2Y_12_ inhibitor, such as clopidogrel or prasugrel, has become a standard therapy in patients at risk of thrombotic vascular events [1–4]. The rationale of this approach is to target the primary drivers of platelet aggregation: thromboxane A_2_ (TXA_2_) and ADP. Aspirin reduces platelet TXA_2_ production and P2Y_12_ receptor blockers prevent binding of the ligand ADP. The observation that strong P2Y_12_ inhibition alone can reduce TXA_2_-dependent platelet aggregation in both *in vitro* and *in vivo* models [5–8] has raised the question of whether aspirin provides an additional anti-platelet effect in the presence of strong P2Y_12_ receptor blockade [9, 10]. In addition to its anti-platelet effects, aspirin also reduces the formation of other intravascular prostanoids, such as the vasoprotective hormone prostacyclin (PGI_2_) [11]. PGI_2_ may be important in controlling platelet responses to pro-aggregatory stimuli and we have proposed that the administration of aspirin in the setting of strong P2Y_12_ receptor blockade removes this protective factor, while the reduction of TXA_2_ adds little to the anti-platelet effect [9]. The production of each prostanoid within the body can be assessed by measurement of the stable urinary metabolites: 2,3-dinor-6-keto PGF1_α_ (PGI-M) for PGI_2_ and 11-dehydro-TXB_2_ (TX-M), for TXA_2_.

In the present study, we aimed to investigate, in healthy male volunteers, whether co-administration of either low or high doses of aspirin with prasugrel provide any additional beneficial effects on platelet reactivity and intravascular prostanoid levels, over those seen with prasugrel alone.

## Methods

### Study participants

In all, 9 healthy male volunteers, aged 18–40 years, were recruited and participated in the present study. The volunteers’ health statuses were determined through their medical histories and physical examination including blood pressure, pulse rate, blood chemistry and urinalysis. Volunteers with normal clinical profiles were included in the study. The study was approved by the St Thomas’s Hospital Research Ethics Committee (Ref. 07/Q0702/24), conducted according to the Declaration of Helsinki and all volunteers gave written informed consent before entering the study.

### Study protocol

Before starting this 21-day study, all volunteers had abstained from aspirin, non-steroid anti-inflammatory drugs (NSAIDs), paracetamol or any other anti-platelet therapy for 14 days. The volunteers received a 60-mg loading dose of prasugrel on day 1 of the study and a maintenance dose of 10 mg prasugrel per day on days 2–21 (Efient®, Eli Lilly and Company, Basingstoke, UK). On day 8 of the study, volunteers began taking 75 mg aspirin and on day 15 the aspirin dose was increased to 300 mg per day until day 21 (Angettes 75®; Bristol-Myers Squibb, Uxbridge, UK). Compliance was assessed by interview. Blood and urine samples were collected on day 0, before commencing drug treatment, and on days 7, 14 and 21 of treatment.

### Blood collection for platelet aggregation studies

Blood was collected by venepuncture into tri-sodium citrate (3.2%, 1:9 v/v; Sigma, Poole, Dorset, UK). Platelet-rich plasma (PRP) was obtained by centrifugation at 175 × *g* for 15 min at 25 °C. Platelet-poor plasma (PPP) was obtained by centrifugation of PRP at 15 000 × *g* for 2 min. All experiments were completed within 2 h of blood collection.

### VerifyNow™ P2Y_12_ and aspirin cartridge assays

Blood was collected by venepuncture into 2-mL partial fill vacuum tubes containing tri-sodium citrate (3.2% final concentration; Griener Bio-One, Stonehouse, UK). VerifyNow™ assays for effects of P2Y_12_ receptor blockade and aspirin activity were performed in accordance with the manufacturer’s instructions (VerifyNow™; Accumetrics, Elitech, UK). A positive response to aspirin was taken as an aspirin response unit (ARU) score < 550, as described in the cartridge package insert.

### 96-well plate light transmission aggregometry

To assess the aggregation of platelets in 96-well plates a modified light transmission method was used. Briefly, 100 μL samples of PRP were placed into the individual wells of a 96-well microtiter plate (Nunc, Lutterworth, Leicestershire, UK) containing 10 μL of vehicle or agonist: ADP (0.1–30 μm; LabMedics, Salford, Manchester, UK), arachidonic acid (AA; 0.03–1 mm; Sigma), Horm collagen (0.1–30 μg mL^−1^; Nycomed, Linz, Austria), epinephrine (0.001–100 μm; LabMedics), TRAP-6 amide specific for PAR1 (SFLLRN; 0.1–30 μm; Bachem, Bubendorf, Switzerland) and the stable TXA_2_-mimetic U46619 (0.1–30 μm; Cayman Chemical Company, Ann Arbor, MI, USA). The plate was then placed into a 96-well plate reader (Tecan Sunrise, Tecan, Reading, UK) at 37 °C, and absorbance was measured at 595 nm every 15 s for 16 min with vigorous shaking between readings. Percentage aggregation was calculated with reference to the absorbance of PPP as a surrogate for 100% aggregation. Graphs shown are for aggregation responses at 16 min.

### Thromboxane B_2_ assay

At the end of the PRP aggregation monitoring (i.e. 16 min), cyclo-oxygenase activity was halted by the addition of 1 mm diclofenac (Sigma), the samples were centrifuged at 1300 × *g* for 10 min at 5 °C, and the supernatants removed and frozen. In samples from PRP stimulated with either arachidonic acid or collagen, plasma thromboxane B_2_ (TXB_2_) levels, as a surrogate for TXA_2_ production, were determined using a selective, competitive EIA (Cayman Chemicals) in accordance with the package insert. In our previous studies we have found readily detectable levels of TXB_2_ after platelet activation by either collagen or AA, but assessments to be less robust for other agonists [7,8,10,12]. Samples were diluted between 1:10 and 1:1000 in diluent and assayed parallel to known TXB_2_ standards and a maximum binding control. The percentage binding of known standards was calculated in reference to the maximum binding (zero TXB_2_) control wells, plotted against the logarithm of concentration and analyzed by non-linear regression using a four-parameter logistical fit model. Unknown samples were expressed in a similar fashion, interpolated from this standard curve and corrected for dilution. All statistical analyzes were performed in Prism v5.0 (GraphPad software, La Jolla, CA, USA).

### Urine collection and storage

A fresh mid-stream urine sample was collected into sterile containers and the preservative chlorhexidine-propyl gallate (1:1, 20% (wt/v) aqueous chlorhexidine digluconate and 20% (wt/v) *n*-propyl gallate in methanol) added at 1:500 parts. Samples were then stored at −40 °C for later assay of urinary prostanoid metabolites. All samples were obtained 3–6 h after dosing on each study day.

### Urinary prostanoid metabolite assays

PGI-M (2,3-dinor-6-keto PGF1_α_) and TXB-M (11-dehydro-TXB_2_) were quantified using stable isotope dilution assays with gas chromatography/mass spectrometry, as previously described [13].

### Stastical analysis

Although the present study included only nine subjects we are confident that this is sufficient to detect differences between the conditions that we have investigated. The variability of platelet aggregation responses between healthy volunteers in this study was 12% for AA, 8% for ADP, 5% for collagen, 8% for TRAP-6 and 6% for U46619 and we propose this low level of variability allowed changes in platelet reactivity to be detected readily, as we have found in previous studies [8,12]. Indeed, power calculations demonstrate that with our sample size (*n* = 9) in healthy volunteers and standard deviation (SD) of response (< 0.45) we had a > 98% power to detect after analysis of data by two-way anova and Bonferroni’s post tests [14]. Similarly, others have detected effects in volunteer groups of 8–12 subjects [15, 16].

All statistical analyzes were conducted using GraphPad Prism v5 (GraphPad). Agonist concentration response curves were plotted and analyzed according to the four parameter logistic equation: Y = Bottom + (Top − Bottom)/[1 + 10^((LogEC50-X) × HillSlope)].

Concentration-response curves were compared using two-way anova and Bonferroni’s post-tests whereas urinary metabolite data were compared by one-way anova with Bonferroni’s post-tests. In each case statistical significance was taken as *P* < 0.05. Other analyzes are stated in text as appropriate.

## Results

### Responses to prasugrel alone and in combination with aspirin in the VerifyNow™ P2Y_12_ and aspirin cartridge assays

**Fig 1:**
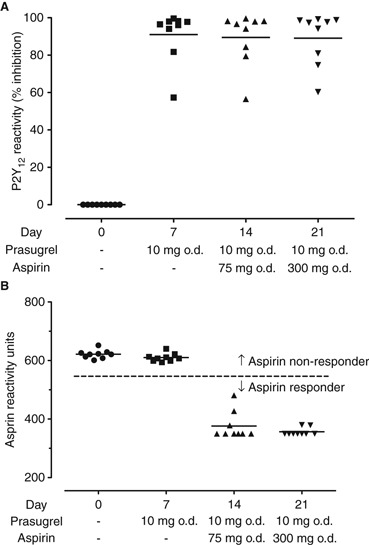
VerifyNow™ aspirin and P2Y_12_ cartridge reactivity. Upper panel, percent inhibition of P2Y_12_ cartridge reactivity, normalized to baseline for values for each of nine study participants. Lower panel, Aspirin Reactivity Units (ARUs) for each of nine study participants at baseline, and study days 7, 14 and 21. ARU values < 550 indicate a response to aspirin (abnormal platelet response to arachidonic acid). In each panel, group means are indicated by a horizontal line. o.d., once daily.

To confirm the response of the study subjects to both prasugrel and aspirin we used the VerifyNow™ cartridge assay system. None of the subjects had positive tests for either P2Y_12_ inhibitors or aspirin at baseline (Fig. 1). With prasugrel alone, eight of the nine study participants had > 95% inhibition of baseline results on the P2Y_12_ assay, whereas the ninth participant had only 50% inhibition on this assay, and these results were maintained with the addition of both low- and high-dose aspirin (Fig. 1A). Although this subject was an outlier from the group, based on this assay, all data relating to this subject were included in further analyzes. It would have been interesting to test the efficacy of the prasugrel active metabolite in a PRP sample of this individual to explore reasons for this difference, but we could not arrange further blood sampling. After the addition of low-dose aspirin (75 mg), all subjects were identified as aspirin responders, and these responses were maintained at the higher dose of aspirin (300 mg) (Fig. 1B). Aspirin responsiveness was determined as an aspirin response units (ARU) score of < 550.

### Effects of prasugrel alone and in combination with aspirin on *ex vivo* platelet reactivity, in 96-well plate aggregometry

**Fig 2:**
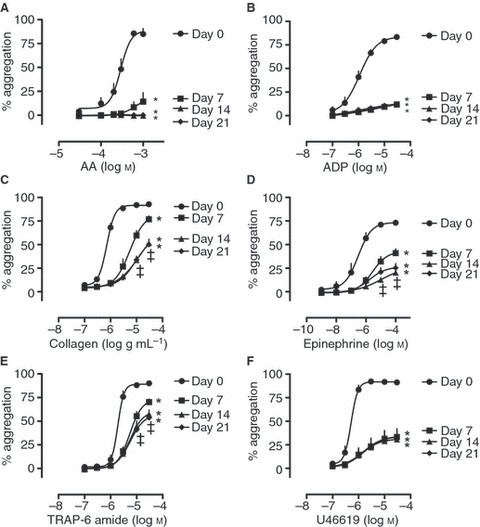
Platelet aggregation induced by arachidonic acid (0.03–1 mm; panel A), ADP (0.1–30 μm; panel B), collagen (0.1–30 μg mL^−1^; panel C), epinephrine (0.001–100 μm; panel D), TRAP6 (0.1–30 μm; panel E) and U46619 (0.1–30 μm; panel F) at baseline and on days 7, 14 and 21 (prasugrel, prasugrel + low aspirin and prasugrel + high aspirin, respectively). Data shown are mean ± standard error of the mean (SEM) of responses measured by 96-well plate aggregometry in citrated platelet-rich plasma (PRP) prepared from nine different individuals. All data points were analyzed by two-way anova with Bonferroni’s post test. *Represents a significant difference, *P*-value < 0.05, compared with baseline; ‡represents a significant difference, *P*-value < 0.05, between prasugrel and prasugrel + low aspirin. Symbols at the end of rows indicate a difference between all points; symbols at individual points signify particular differences.

Platelet aggregation to AA, ADP and U46619 was strongly inhibited at all concentrations by prasugrel alone, and prasugrel in combination with aspirin, although there was no further reduction with aspirin at either dose (Fig. 2, panels A, B and F). Responses to collagen, epinephrine and TRAP-6 amide were also moderately inhibited by prasugrel alone, and at the highest concentrations of these agonists there was a further effect seen with aspirin, although there was no variation between the effects of the two aspirin doses (Fig. 2, panels C, D and E).

### Thromboxane production in response to AA and collagen by 96-well plate aggregometry

**Fig 3:**
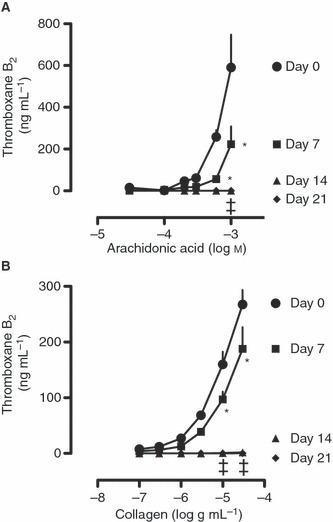
Platelet production of TXA_2_ induced by arachidonic acid (0.03–1 mm; panel A) and collagen (0.1–30 μg mL^−1^; panel B) at baseline and days 7, 14 and 21 (prasugrel, prasugrel + low aspirin and prasugrel + high aspirin, respectively). Data shown are mean ± standard error of the mean (SEM) of responses collected from 96-well plate aggregometry in citrated platelet-rich plasma (PRP) prepared from nine study particpants. *Shows *P* < 0.05 difference from baseline by two-way anova plus Bonferroni’s post test; †Shows *P* < 0.05 difference between prasugrel and prasugrel + low aspirin. Symbols at the end of lines signify a difference in set; symbols at individual points signify particular differences.

Prasugrel administration reduced the production of TXA_2_ in response to both AA and collagen by platelets in 96-well aggregometry (Fig. 3A and B, respectively). Co-administration of either dose of aspirin with prasugrel resulted in the complete abolition of any remaining TXA_2_ production.

### Production of urinary prostanoid metabolites

In addition to reducing platelet-derived TXA_2_ production, aspirin can, by inhibition of cyclooxygenases, reduce the production of vascular PGI_2_. To assess the changes in urinary prostanoid levels we studied the stable urinary metabolites of thromboxane, 11-dehydro-TXB_2_ (TX-M), and PGI_2_, 2,3 dinor-6-keto-PGF1_α_ (PGI-M), at each time point of the study.

**Fig 4:**
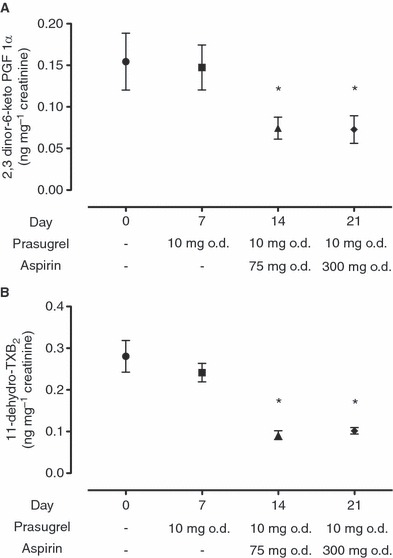
Urinary PGI-M (panel A) and TX-M (panel B) production at days 0, 7, 14 and 21 (baseline, prasugrel, prasugrel + low aspirin and prasugrel + high aspirin, respectively). Values for urinary prostanoid metabolites (PGI-M or TX-M) are corrected against urinary creatinine concentration. Data shown are mean ± standard error of the mean (SEM) of nine study participants. *Shows *P* < 0.01 difference from Day 0 by one-way anova plus Bonferroni’s post test.

Neither urinary PGI-M nor TX-M were reduced from baseline values with prasugrel alone (Fig. 4, panel A and B, respectively, *P* > 0.05). When prasugrel and low-dose aspirin were co-administered, the levels of both urinary prostanoid metabolites decreased (Day 0 or Day 7 vs. Day 14, PGI-M, *P* < 0.01; TX-M, *P* < 0.001), although there was no further decrease seen with prasugrel in combination with high-dose aspirin (Day 14 vs. Day 21, PGI-M, *P* > 0.05; TX-M, *P* > 0.05).

## Discussion

In this healthy volunteer study we looked at the effects of a strong P2Y_12_ receptor blocker, prasugrel, alone and in combination with low- and high-dose aspirin, on platelet reactivity and urinary prostanoid metabolites. After a standard dosing protocol, prasugrel inhibited platelet aggregation to a range of agonists, with additional effects of aspirin seen only against the highest concentrations of collagen, epinephrine and TRAP-6. The urinary levels of TXA_2_ and PGI_2_ metabolites were unaffected by prasugrel alone, but were both reduced by aspirin. Higher doses of aspirin in combination with prasugrel did not have any additional effects on either platelet reactivity or urinary prostanoid metabolites, compared with those seen with prasugrel and low-dose aspirin.

The effects of prasugrel alone, and in combination with aspirin, were similar to those seen in a recent *in vitro* study reported by our group [10], although we observed a slightly larger effect of aspirin *ex vivo*. The observations presented here confirm that P2Y_12_ receptor blockade by prasugrel strongly inhibits platelet aggregation to ADP as measured by 96-well plate aggregometry, although a small residual response was seen to the highest concentrations of this agonist. In addition, platelet production of TXA_2_ and TXA_2_ (TP) receptor-dependent platelet aggregation was reduced. In contrast to our recent *in vitro* findings [10], TP receptor-dependent aggregation was not entirely abolished by prasugrel. As TP-mediated platelet aggregation relies upon the ADP-P2Y_12_ axis [5, 6, 10] this may be explained by the residual responses to ADP observed in the present study. Alternatively, this may reflect a P2Y_12_-independent response to TP receptor stimulation, although it is unclear why this may be apparent *ex vivo* after prasugrel treatment, although not after *in vitro* treatment with prasugrel active metabolite.

When aspirin is used in combination with prasugrel there is a complete inhibition of platelet TXA_2_ production, although the further reduction of platelet aggregation, compared with prasugrel alone, is limited. For instance, the abolition of TXA_2_ in collagen-induced aggregation results in a further reduction of platelet aggregation only at high concentrations of this agonist. Aspirin also has a small additional effect on the platelet aggregation induced by strong PAR1 activation (by TRAP-6 amide; SFLLRN) and the highest concentrations of epinephrine. In these circumstances, it is likely that the effect of TXA_2_ (that is lost by the addition of aspirin) is partly mediated by the residual potential for TP receptor-induced platelet aggregation, as described above.

Although the combination of prasugrel and aspirin severely limits the platelet aggregation response to soluble mediators (ADP and TXA_2_) there is still a reasonable aggregation response to high concentrations of platelet agonists that act through different signaling pathways. For instance, PAR1 activation by TRAP-6 amide is able to induce approximately 50% aggregation, even when ADP is rendered inactive and TXA_2_ formation is inhibited. Additionally, high concentrations of collagen induced a moderate level of aggregation in the absence of soluble mediator activities. This is consistent with strong signaling from the G-protein coupled receptors, a topic that has been addressed in two informative reviews [17, 18], and from platelet integrins [19]. Measurable residual platelet reactivity is unsurprising given the wealth of clinical data that reports the occurrence of occlusive thrombotic events in patients taking both aspirin and P2Y_12_ receptor blockers [20–23].

The predominant choice of which aspirin dose to use in dual anti-platelet therapy varies with geographic location [24] and so it was useful to include both a low- and a high dose of aspirin in combination with prasugrel in the present study. Consistent with meta-analysis of aspirin as a monotherapy, which show that the efficacy of aspirin is not enhanced at higher doses [1], there was no evidence that the higher dose of aspirin produced any further anti-platelet effects than a strong P2Y_12_ inhibitor together with low-dose aspirin. The present study did not determine the effects of aspirin alone on platelet aggregation using the 96-well plate assay method, although previous work by our group has demonstrated that this assay readily detects the effects of aspirin in healthy volunteers [12].

In addition to platelet reactivity, we measured the urinary metabolites of both TXA_2_ and PGI_2_, which are considered a marker of *in vivo* prostanoid levels [11]. Urinary metabolites were measured within 6 h of dosing on the relevant study day, which is within the time period in which the effects of aspirin are maintained in healthy subjects taking consecutive daily doses of this drug [11,25]. In healthy volunteers PGI-M and TX-M were unaffected by prasugrel alone, although these results contrast with the effects of clopidogrel, which we have previously reported in a similar study [8]. Urinary TX-M formation was reduced after 7 days of clopidogrel dosing in healthy volunteers, although in this case the samples were measured by enzyme immunoassay rather than mass spectroscopy [8] and we have greater confidence in the data we present here; although the small number of individuals included in each study makes a definitive analysis difficult, especially because of the large inter-individual variability in urinary PGI-M and TX-M levels. The failure of prasugrel to reduce TX-M excretion seems contradictory to the reduction of *ex vivo* TXA_2_ formation that is associated with clopidogrel [8,26] and now prasugrel; however, TX-M is used as a marker of physiological TXA_2_ levels whereas *ex vivo* TXA_2_ formation describes the production of this lipid mediator in response to strong thrombotic stimuli, which are absent in young healthy volunteers. In patients with vascular inflammation [13] and atherosclerosis [27], urinary TXA_2_ formation is increased and the effects of prasugrel on urinary TX-M may be different in these pathologic states where *in vivo* platelet activation may contribute more to urinary TX-M levels.

In the present study, both prostanoids were reduced, independently of dose, when aspirin was co-administered. It has been previously suggested in a similarly sized study that the reduction of PGI-M by aspirin is dose dependent, although when scrutinizing these earlier data we found a large overlap between the values for groups taking 75 and 325 mg aspirin similar to the results we report here [11]. It is unclear from our data but it is possible that in larger studies we may have seen a dose-dependent relationship between aspirin and urinary PGI-M.

The effect of vascular-derived PGI_2_ cannot be observed in *ex vivo* aggregation studies owing to the short half-life of this prostanoid. However, *in vitro* studies suggest that in the presence of strong P2Y_12_ receptor blockade platelets may be more sensitive to the inhibitory effects of this hormone [28], and it is plausible that such a phenomenon has the potential to contribute to the efficacy of drugs targeting the P2Y_12_ receptor pathway. Clearly, this can only occur if the production of vascular prostacyclin is preserved, whereas the co-administration of aspirin with P2Y_12_ receptor blockers results in reduced PGI_2_ formation and a potential limitation of the anti-thrombotic effect. Furthermore, loss of vascular PGI_2_ has been proposed as the underlying mechanism of cardiovascular risk that is associated with NSAIDs based on data derived from urinary PGI-M measurements [29].

A limitation of the present study was the continuous administration of treatments without any ‘wash-out’ periods to differentiate the effects of each drug combination from a potential effect of time. An alternative study design could have also included a treatment period of aspirin alone. Clearly, such a study would have been a much larger undertaking and would have provided further levels of information. However, it is our belief that for the mechanisms of drug action that we wished to explore the study design chosen was appropriate.

In conclusion, the present study indicates that strong P2Y_12_ receptor blockade produces an inhibition of platelets that is only slightly enhanced by aspirin, as assessed *ex vivo*. Aspirin also reduces the levels of PGI_2_*in vivo*, an effect that is suggested to increase the risk of thrombosis. As no clinical trial has tested the effects of aspirin in the presence of strong P2Y_12_ receptor blockade it is unclear whether or not aspirin enhances or limits the anti-thrombotic effect that can be achieved with drugs such as prasugrel. We would therefore suggest that dedicated clinical trials should be considered to ascertain the true benefits, and rates of adverse events, when using aspirin alongside an effective, strong P2Y_12_ receptor blocker.
